# Low density lipoprotein modified silica nanoparticles loaded with docetaxel and thalidomide for effective chemotherapy of liver cancer

**DOI:** 10.1590/1414-431X20176650

**Published:** 2018-03-01

**Authors:** Man Ao, Xu Xiao, Yazhou Ao

**Affiliations:** 1 Department of Oncology, Affiliated Hospital of Chengde Medical University, Chengde, Hebei, China; 2 Department of Pharmacy, Affiliated Hospital of Chengde Medical University, Chengde, Hebei, China; 3 Department of Thyroid Surgery, Affiliated Hospital of Chengde Medical University, Chengde, Hebei, China

**Keywords:** Docetaxel, Thalidomide, Low density lipoprotein, Silica nanoparticles, Liver cancer

## Abstract

In the present study, we successfully developed a docetaxel (DTX) and thalidomide (TDD) co-delivery system based on low density lipoprotein (LDL) modified silica nanoparticles (LDL/SLN/DTX/TDD). By employing the tumor homing property of LDL and the drug-loading capability of silica nanoparticles, the prepared LDL/SLN/DTX/TDD was expected to locate and specifically deliver the loaded drugs (DTX and TDD) to achieve effective chemotherapy of liver cancer. *In vitro* analysis revealed that nano-sized LDL/SLN/DTX/TDD with decent drug loading capabilities was able to increase the delivery efficiency by targeting the low density lipoprotein receptors, which were overexpressed on HepG2 human hepatocellular liver carcinoma cell line, which exerted better cytotoxicity than unmodified silica nanoparticles and free drugs. *In vivo* imaging and anti-cancer assays also confirmed the preferable tumor-homing and synergetic anti-cancer effects of LDL/SLN/DTX/TDD.

## Introduction

Inorganic materials, such as calcium carbonate nanoparticles (1), gold nanoparticles (2) and silica nanoparticles (3), have attracted the interests of many studies. A milestone was the introduction of silica nanoparticles (SLNs) over 50 years ago. The numerous advantages of SLNs, such as high biocompatibility and biodegradability, have made them suitable carriers for biomedical applications (4). Moreover, due to their large surface area and pore volume, they also possess high drug loading capacity for a variety of drugs ranging from hydrophobic ones to hydrophilic ones, which at the same time capable of further being employed as versatile carriers for drug delivery and imaging applications (5).

In order to effectively act in cancer cells, carriers require the ability to bypass the multiple extracellular and intracellular barriers in the body, besides having a high loading degree (6). Two of the greatest challenges are to escape capture by the reticuloendothelial system (RES) and to target the tumor tissue (7). The *in vivo* fate of SLNs is somehow predictable since bare SLNs with raw surface properties are usually treated as foreign materials that can be readily captured and excreted by RES (8). To overcome this issue, lipid matrix-encapsulated SLNs that could provide a tunable layer to seal the surface of SLNs have been reported. The chemical structure of such layer has been shown to decrease their interactions, which is beneficial to the *in vivo* performance of SLNs (9). In addition, in order to increase their accumulation at the tumor tissue, tumor-targeting ligands were further added to construct a multifunctional system (10). The preliminary requirement for tumor-targeting ligands is that they specifically recognize the abnormal upregulated receptors on the surface of cancer cells to guide the drug delivery system (DDS) to the wanted tumor tissue (11).

The expression of low density lipoprotein receptors (LDLR) has been proven to be upregulated on the surface of many cancer cell lines, including breast cancer, prostate cancer and liver cancer (12–14). This provides an alternative way for scientists to specifically target the required cancer cells. Low density lipoprotein (LDL) is the dominant ligand for the recognition of LDLR (15). On the other hand, LDL is also the major cholesterol transporter in the plasma. As an endogenic component within the human body, LDL shows high biocompatibility and extremely low cytotoxicity, being widely adopted in DDS (16). The LDL-modified nanoparticles not only have high biocompatibility and low cytotoxicity, but also tumor-targeting capabilities. This suggests that drug carriers with surface-modified LDL may be used as a suitable system for chemotherapeutic agent carriers to neoplastic cells (17,18).

We recently discovered that the combination of docetaxel (DTX) and thalidomide (TDD) would be beneficial for cancer therapy. As TDD is a common drug for protection of liver injury (19), we thereby want to combine these two drugs in a tumor-targeting DDS to test a new strategy for hepatocellular liver carcinoma therapy. We developed LDL-modified lipid silica nanoparticles to co-deliver DTX and TDD, with the aim to construct a DDS (LDL/SLN/DTX/TDD) capable of delivering a both DTX and TDD specifically to the tumor site to achieve a better anti-cancer effect compared with bare SLNs and free drugs.

## Material and Methods

### Material

Triton X-100, tetraethyl orthosilicate (TEOS) and N-(2-aminoethyl)-3-aminopropyltrimethoxysilane (AEAPS) were obtained from the Sinopharm Chemical Regent Co., Ltd (China). DTX and TDD were supplied by Aladdin Bio-chem Technology (China). Plasma-derived LDL was obtained from Intracel (USA). 3-(4,5-dimethylthiazol-2-yl)-2,5-diphenyl tetrazolium bromide (MTT), coumarin 6 and DiR iodide (DiR) were purchased from Sigma-Aldrich (USA). All other chemicals and reagents otherwise stated were from Sinopharm Chemical Reagent Co., Ltd. (China) and of analytical grade.

### Cell culture and animal model

Human liver cancer cell line HepG2 was a gift from American Type Culture Collection (ATCC, USA). Cells were maintained in Dulbecco minimum essential medium (DMEM) (containing 10% volume ratio of endotoxin-free fetal bovine serum, 2 mM glutamine, 100 U/mL streptomycin and penicillin; Gibco, USA) at 37°C in a humid atmosphere with 5% CO_2_ and 95% air.

Male Balb/c nude mice (∼20 g) were acquired from Suzhou Belda Bio-Pharmaceutical Co. (China) and maintained in specific pathogen-free lab at the homothermal condition of 25±2°C with free access to food and water. All procedures were approved by the ethics committee of Affiliated Hospital of Chengde Medical University. The generation of HepG2 tumor bearing nude mice model was based on a previous report (11). Briefly, 2×10^6^ HepG2 cells were dispersed in 100 μL of serum-free DMEM and then subcutaneously injected into the flank of mice. Mice with tumor volumes at about 100 mm^3^ were recruited and randomly assigned to perform *in vivo* experiments.

### Preparation of LDL/SLN/DTX/TDD

Amine-decorated SLN was firstly synthesized in a water-in-oil microemulsion with minor modification as previously reported (20). In brief, a water-in-oil microemulsion was prepared by mixing 1.8 mL of Triton X-100, 1.6 mL of n-hexanol and 7.5 mL of cyclohexane. The mixture was gently agitated for 0.5 h to obtain a transparent solution. Afterwards, 180 μL TEOS together with 60 μL AEAPS were added under agitation to allow for well dispersion within the microemulsion as precursors for silica formation. One hundred microliter of NH_4_OH was then added to initiate the polymerization process. The reaction was performed continuously under room temperature for 24 h, followed by addition of 50 mL of ethanol to precipitate the amine-decorated SLN. The precipitate was washed with ethanol and water alternatively several times to remove the surfactant and unreacted reactants from the particles.

The prepared amine-decorated SLN was resuspended in 10 mL of mixed solution of ethanol and pyridine (v/v 1:1) with agitation. DTX dissolved in ethanol (5 mg/mL) and TDD dissolved in pyridine (5 mg/mL) were added and co-cultured with amine-decorated SLN for 30 min. Afterwards, the drug-loaded amine-decorated SLN was isolated under high speed centrifugation (8000 *g*, 10 min, 25°C, CR21, Hitachi, Japan). The remaining DTX and TDD in the supernatant was determined using high performance liquid chromatography (HPLC, Agilent 1300 series, USA). The conditions for DTX were: Diamond C18 column (4.6×150 mm, USA) at a constant temperature of 25°C; the mobile phase consisted of a mixture of acetonitrile (TEDIA, USA) and deionized water (Millipore, USA) at the volume ratio of 3:2; the applied flow rate was 1 mL/min with a spectrophotometric detection set at 230 nm. For TDD the conditions were: Waters Nova-Pak C18 column (3.9×150 mm, USA) at a constant temperature of 40°C; the mobile phase consisted of deionized water, acetonitrile and phosphoric acid at the volume ratio of 85:15:0.1; the applied flow rate was 2 mL/min with a spectrophotometric detection set at 237 nm.

The drug-loaded SLN was resuspended in aqueous solution, to which LDL (1 mg/mL) was added and co-incubated with gentle agitation at room temperature for 6 h. Finally, the LDL/SLN/DTX/TDD was isolated from the solution using high speed centrifugation (8000 *g*, 10 min, 25°C).

Drug loading content (DLC) was calculated according to the following formula: DLC (wt%) = (weight of loaded drug/weight of LDL/SLN/DTX/TDD)×100%.

### Particle size and zeta potential measurement

Characterization of nanoparticles concerning their size distributions, polydispersity index (PDI) and zeta potential were assessed at 25°C by dynamic light scattering (DLS) and electrophoretic light scattering methods using a Zeta plus zeta potential analyzer (Brookhaven Instruments Corp., USA).

### *In vitro* release experiments

The release behavior of DTX and TDD from LDL/SLN/DTX/TDD was investigated. LDL/SLN/DTX/TDD was diluted in phosphate buffer saline (PBS, pH 7.4 and 5.0, containing 0.1% Tween 80, w/v) and maintained at 37°C with gentle shaking (100 rpm). At predetermined time intervals, 1 mL of the solution was extracted and equal volume of fresh medium was supplied. The extracted solution was centrifuged at 8000 *g* for 10 min at 25°C to remove the nanoparticles and the supernatant was measured by HPLC to determine the DTX and TDD content.

### Cellular uptake of LDL/SLN/DTX/TDD

Fluorescent probe coumarin 6 (C6) was dissolved in ethanol (0.1 mg/mL) and loaded into nanoparticles along with drug loading. The internalization profile of SLN/DTX/TDD and LDL/SLN/DTX/TDD in HepG2 cell line was assessed by monitoring the fluorescence signal of C6. HepG2 cells cultured in confocal dishes (Φ=15 mm) with 60% confluence were treated with 1 mL of the serum-free medium containing free C6, C6 containing SLN/DTX/TDD or C6 containing LDL/SLN/DTX/TDD at the C6 concentration of 300 ng/mL. After 2, 4 and 6 h of incubation, the medium was discarded and cells were rinsed three times with PBS to remove the remaining nanoparticles. Afterwards, cells were treated with trypsin to obtain mono dispersed cell suspension. For quantitative determination of the fluorescence intensity of each group, the culture media were discarded, cells were harvested and subjected to flow cytometer (FCM, BD FACSCalibur™, USA). Excitation was conducted with a 485 nm argon laser, and the fluorescence emission at 595 nm was measured; a minimum of 1×10^4^ cells from each sample were randomly selected for measurement. The potential LDLR-mediated uptake of LDL/SLN/DTX/TDD was confirmed by competitive binding experiments. Briefly, all cells were firstly treated with serum-free medium containing 200 μg/mL of LDL solution for 2 h. Afterwards, free C6, C6 containing SLN/DTX/TDD or C6 containing LDL/SLN/DTX/TDD was added to the same medium to achieve the same C6 concentration as mentioned above. After the same procedure, the fluorescence intensity of each group was quantitatively determined by FCM and compared with LDL untreated ones.

### Cytotoxicity activity

To study the cytotoxicity of free nanoparticles and LDL/SLN/DTX/TDD, the HepG2 cells were detached using 0.5% trypsin, harvested and seeded onto a 96-well plate, and allowed to grow overnight to reach confluence of 70–80%. After that, cells were incubated with fresh medium containing different samples: free DTX solution, free TDD solution, drug-free LDL/SLN, DTX-free LDL/SLN/TDD, TDD-free LDL/SLN/DTX and LDL/SLN/DTX/TDD (DTX and TDD concentration was set at 1, 2, 5, 10, 15, 25 μg/mL). After different intervals of incubation, standard MTT assay was applied to evaluate the cell viability of all tested samples as reported previously (21).

### *In vivo* tumor-targeting of LDL/SLN/DTX/TDD

DiR as a near infrared fluorescent probe was encapsulated into the nanoparticles similar to the C6 loading process. Afterwards, LDL/SLN/DTX/TDD and SLN/DTX/TDD were injected into the HepG2 tumor-bearing mice via tail vein at the DiR dosage of 10 μg/per mouse. The *in vivo* real-time biodistribution of different nanoparticles at 1, 3 and 6 h were recorded using *In vivo* Imaging System (IVIS Lumina LT, Perkin Elmer, USA) with filter sets at excitation and emission of 720 and 790 nm, respectively. In order to confirm the LDLR-mediated targeting capability of LDL/SLN/DTX/TDD, mice were firstly intratumorally injected with LDL (5 mg/kg) 1 h prior to the injection of DiR-loaded LDL/SLN/DTX/TDD and *in vivo* imaged as mentioned above. The tissue distribution of nanoparticles at the end of experiments was assessed by excising the tumor tissues as well as major organs from the sacrificed mice and subjecting samples to *ex vivo* imaging using the same equipment.

### *In vivo* antitumor efficacy

The *in vivo* antitumor efficacy of LDL/SLN/DTX/TDD was further confirmed by employing HepG2 tumor xenograft models. All mice were randomly divided into 5 groups (n=5): 1) saline (control); 2) free DTX; 3) free TDD; 4) SLN/DTX/TDD; 5) LDL/SLN/DTX/TDD. Protocols including administration route and dosing frequency were similar to previous report with some modifications (22). In brief, all formulations were administrated via tail vein (10 mg/kg DTX and/or 25 mg/kg TDD per mouse) once every two days for 7 times. The body weights and tumor sizes of all treated mice were monitored and recorded before injection. Two days after the last injections, three mice in each group were randomly picked and sacrificed. Their tumor tissues were sliced, subjected to hematoxylin and eosin (HE) staining and pictured by microscope (Leica, DM IL LED, Germany).

## Results and Discussion

### Particle size, dispersity, morphology and drug loading of LDL/SLN/DTX/TDD

**Figure 1.**
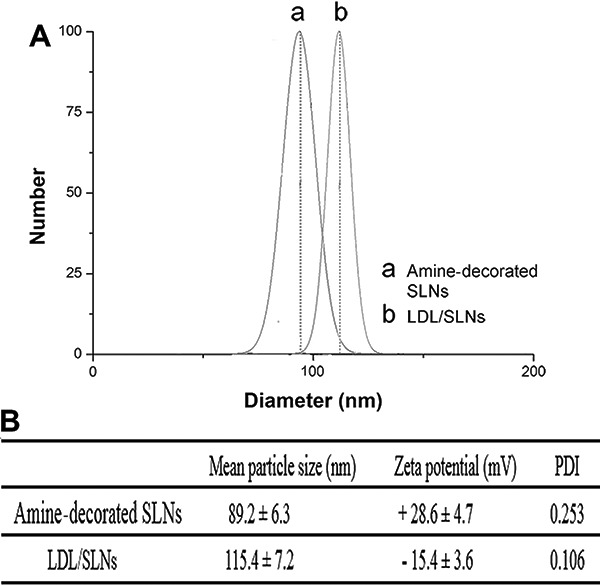
Particle size distribution (*A*) of amine-decorated silica nanoparticles (SLNs) and low density lipoprotein (LDL)/SLNs. Mean particle size, zeta potential and poly dispersion index (PDI) measurements (*B*) of amine-decorated SLNs and LDL/SLNs. Data are reported as means±SD (n=3).

The amine-decorated SLNs with free primary amine groups on their surface can serve as a powerful tool in the assembly of multifunctional DDSs via either chemical reaction or physical interaction (23). The particle size of amine-decorated SLNs were firstly measured by DLS technic. As shown in Figure 1A, the amine-decorated SLNs obtained by water-in-oil microemulsion were nano-sized particles with a diameter of around 89.2 nm. These particles were well dispersed with a relatively small PDI of 0.253. It has been reported that the water-in-oil microemulsion method can control the size of the particles using the well-formed water pools in the microemulsion. These pools are composed of a water core and a surfactant shell which can define the reaction zone. On the other hand, the surfactant shell can also serve as a spacer to prevent the neighboring nanoparticles from aggregation (24,25). The LDL was anchored to the surface of amine-decorated SLNs via electrostatic adsorption. The attachment of LDL resulted in an increase in particle size of LDL/SLNs compared to unmodified SLNs.

Compared to the particle size of amine-decorated SLNs, LDL/SLNs demonstrated a slightly increased size of around 115.4 nm with a decreased PDI of 0.106. The decreased PDI value of LDL/SLNs indicated that surface modification of hydrophilic LDL might further benefit the dispersion of LDL/SLNs since it has been demonstrated by previous reports that hydrophilic surface modification, such as polyethylene glycol (PEG), can increase the colloidal stability of the recipient (26,27). On the other hand, the successful modification of LDL to the surface of SLNs was further proven by zeta potential measurement.

It can be seen from Figure 1B that the originally prepared amine-decorated SLNs are positively charged particles with a surface charge of +28.6 mV. However, the surface charge was totally reversed after modification of LDL with a negative charge of -15.4 mV being observed. According to a previous report, the negatively charged surface might be beneficial for the DDS to bypass the recognition of many active components within the circulation system and ensure safe delivery of the drugs (6).

Due to their large surface area and pore volume, SLNs were capable of loading a variety of drugs ranging from hydrophobic ones to hydrophilic ones with a relatively high loading efficiency. This is beneficial for SLNs to act as a co-delivery carrier for the loading of both DTX and TDD. According to our HPLC analysis, the DLC for DTX and TDD was 15.6 and 10.3%, respectively, which is high enough for both the following *in vitro* and *in vivo* experiments.

### *In vitro* drug release

One fatal drawback of some currently available DDSs is that they are not able to preserve the encapsulated drugs safely in the circulation system. The leaked drugs might cause severe side effects to normal tissues and organs that would lead to a failed chemotherapy.

**Figure 2.**
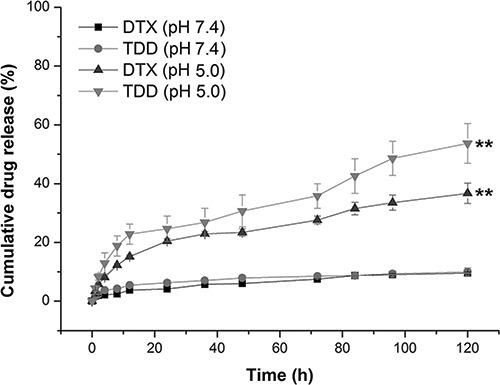
*In vitro* docetaxel (DTX) and thalidomide (TDD) of the drug release system LDL/SLN/DTX/TDD at different pH values (7.4 and 5.0). Data are reported as means±SD (n=3). LDL: low density lipoprotein; SLN: silica nanoparticles. **P<0.01 *vs* pH 7.4 (two-tailed Student's *t*-test).

In order to simulate the drug release profile of LDL/SLN/DTX/TDD in physiological and neoplastic conditions, the drug release percentages of our system were monitored and recorded under PBS with different pH values. The pH 7.4 mimicked the physiological condition while pH 5.0 mimicked the intracellular condition (endo-lysosomes) of cancer cells. It can be observed in Figure 2 that the drug release speed of both DTX and TDD was slow in physiological condition (pH 7.4) with less than 10% of the encapsulated drugs being leaked into the medium. This is beneficial for the safe and effective delivery of both DTX and TDD to the neoplastic cells since it usually takes 12 to 24 h for DDSs to accumulate at the tumor tissues (28,29). On the contrary, under the acidic condition, pH 5.0, the drug release of both DTX and TDD was accelerated. The release rate of DTX was more than three time the speed of that in pH 7.4, with 36.7% of the encapsulated drugs being released after 120 h of incubation. On the other hand, it is worth noting that the release speed of TDD was further accelerated as almost 53.7% of the drugs was released at the same time. This can be explained by the fact that TDD is a weak base, the solubility of which might be increased in acidic conditions, increasing the drug release of the loaded TDD under acidic conditions.

These results were encouraging since they reveal the potential of LDL/SLN/DTX/TDD to accelerate its drug release under acidic condition while maintaining the cargo in normal conditions. It has been reported that tumor tissue is composed of many highly active cells with high expression of many enzymes and capable of secreting various constituents. Thus, the drug release speed of LDL/SLN/DTX/TDD is expected to be further enhanced because the decomposition of the carrier as well as the competitive dissociation under such condition will be more serious than that in PBS. This could be beneficial for the selective drug release in neoplastic tissues and not in normal areas, to reduce the side effects of the chemotherapy and increase the anti-cancer efficacy of the drugs.

### Cellular uptake of LDL/SLN/DTX/TDD

It has been demonstrated by many previous articles that LDL anchored toward the surface of the DDS can target LDLR (30,31), which is excessively expressed in various cancer cells, including liver cancer (14). In order to verify that our DDS was also capable of specifically targeting LDLR-overexpressed HepG2 cells, C6 was employed as both a fluorescence probe and a hydrophobic drug molecule that mimic the uptake profile of DTX and TDD, followed by quantitative analysis of the uptake behavior of different samples by FCM at different time points.

**Figure 3.**
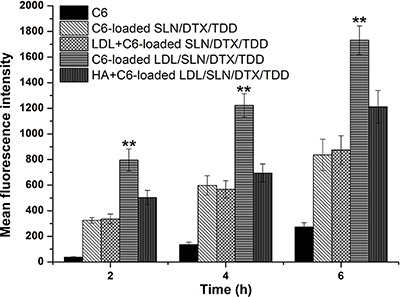
*In vitro* quantitative flow cytometric analysis of free coumarin 6 (C6), C6-loaded SLN/DTX/TDD and C6-loaded LDL/SLN/DTX/TDD with and without low density lipoprotein (LDL) pretreatment in HepG2 cells for 2, 4, and 6 h of incubation. Data are reported as means±SD (n*=*3). SLN: silica nanoparticles; DTX: docetaxel; TDD: thalidomide; HA: hyaluronic acid. **P<0.01 *vs* LDL pretreated groups (two-tailed Student's *t-*test).

As shown in Figure 3, higher C6 fluorescence signals were observed at 2 h post incubation in the cells of LDL/SLN/DTX/TDD group, compared to that of LDL unmodified SLN/DTX/TDD, as revealed by FCM data. This difference did not disappear but became more pronounced as incubation continued. It was also observed from FCM data that the fluorescence intensity of LDL/SLN/DTX/TDD was approximately 2.07-fold higher than that of SLN/DTX/TDD after incubation for 6 h, suggesting that LDL/SLN/DTX/TDD can be more effectively internalized by HepG2 cells than its counterpart SLN/DTX/TDD, possibly via the LDLR mediated endocytosis pathway.

It was also noted that, during the whole incubation time, the fluorescence signals in free C6 treated cells were much lower than that of the DDSs treated groups. This could be explained by the fact that C6 as a drug molecule can be excreted outside the cells via P-glycoprotein, a common transporter that is associated with multi-drug resistance of cancer cells (32). However, some evidence has shown that DDSs-based delivery can bypass and overcome such excretion effect to improve the anti-cancer efficiency of the loaded drugs.

In addition, we also performed competitive uptake experiments to further confirm the LDLR associated uptake of LDL/SLN/DTX/TDD. A great decline in fluorescence intensity of LDL/SLN/DTX/TDD group was observed at all time intervals after pretreatment with excess LDL while the fluorescence intensity of SLN/DTX/TDD group still remained at the same level, which was in line with a similar report that showed that a competitive molecule could inhibit the uptake of modified DDS to reveal its potential pathways (33,34). These results clearly demonstrated that LDL/SLN/DTX/TDD was internalized into cells via LDLR-mediated endocytosis.

### Cytotoxicity assay

**Figure 4.**
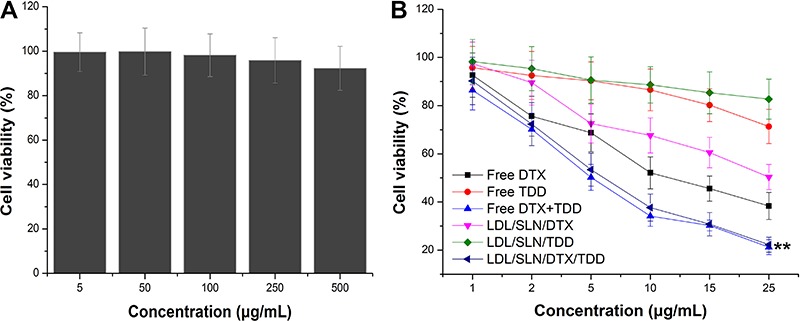
*A*, Cytotoxicity of free LDL/SLNs after 48 h incubation with HepG2 cells. *B*, Cytotoxicity of free DTX, free TDD, LDL/SLN/DTX, LDL/SLN/TDD and LDL/SLN/DTX/TDD against HepG2 cells after 48 h incubation. LDL: low density lipoprotein; SLN: silica nanoparticles; DTX: docetaxel; TDD: thalidomide. Data are reported as means±SD (n*=*3). **P<0.01 *vs* LDL/SLN/DTX or LDL/SLN/TDD (two-tailed Student's *t-*test).

In order to further explore and verify the *in vitro* DTX and TDD delivery efficiency and anti-cancer efficiency of the well-designed LDL/SLN/DTX/TDD, MTT assay was employed to evaluate cell viability and to reflect results of cytotoxicity. Prior to the MTT assays of drug loaded formulations, drug free LDL/SLNs were first conducted with nanoparticle concentrations ranging from 5 to 500 µg/mL to determine whether the carriers we adopted in this study had cytotoxicity effects on HepG2 cells and to what extent it can influence the final results. As shown in Figure 4A, when treated by drug-free LDL/SLNs, no significance was found among all adopted concentrations as more than 90% of the cells survived all the dosing concentrations. This indicated that the LDL/SLNs we constructed had low cytotoxicity and were biocompatible, which thus grant a broad range for their potential application in the field of cancer therapy and other biomedical fields.

The following anticancer assay with drug-loaded nanoparticles showed some interesting results. It was clear that both DTX and TDD had anti-cancer effects on HepG2 cells and this effect was dose-related as higher drug dosing would lead to increased cell mortality. LDL modification can increase the cellular uptake of LDL/SLNs as proven by cellular uptake experiments, as we had expected; the cytotoxicity of cells treated with LDL modified formulations were more severe than the unmodified ones under the same conditions. Moreover, it was interesting to find that although LDL modification can increase the cellular uptake of LDL/SLNs, the cytotoxicity of single drug-loaded formulations have impaired anti-cancer effect compared to free drugs. We speculated that this might be due to the incomplete drug release in HepG2 cells.

In cancer therapy, it is known that drug-loaded DDS cannot compete with free drugs because drug release within DDS cannot reach 100% and, as a result, the cell viability of DDS is higher than that of free drugs (35,36). However, when applied *in vivo*, DDS show more advantages than free drugs as they can be modified to more specifically target tumor tissue and increase local drug concentration as well as avoid side effects (37,38). However, the co-delivery of DTX and TDD exerted much more potent anti-cancer effects than either single drug-loaded formulation or free drugs at all the adopted drug concentrations. At drug concentration of 5 μg/mL, co-delivery of DTX and TDD exerted synergetic effects resulting in a better anti-cancer effect than free drugs at the highest concentration of 25 μg/mL, which indicated that the combination of DTX and TDD might be much more potent in chemotherapy than applying either one alone.

### *In vivo* imaging of LDL/SLN/DTX/TDD

**Figure 5.**
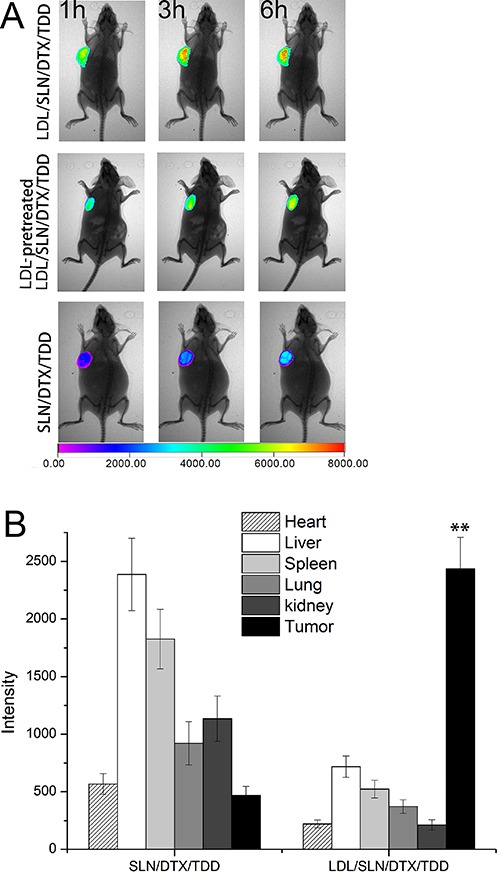
*A*, *In vivo* time-dependent tumor-targeting images after intravenous B injection of DiR-loaded nanoparticles in HepG2 tumor-bearing mice. *B*, Representative *ex vivo* mean fluorescence intensity of dissected tumors and major organs at 6 h post-injection. LDL: low density lipoprotein; SLN: silica nanoparticles; DTX: docetaxel; TDD: thalidomide. Data are reported as means±SD (n*=*3). **P<0.01 *vs* SLN/DTX/TDD (two-tailed Student's *t-*test).

LDL modification on the surface of LDL/SLN/DTX/TDD was expected to aid the nanoparticles to bypass the RES and result in their increased accumulation at the tumor site. To verify this conjecture, we employed non-invasive near infrared optical imagining technique as well as near infrared fluorescence probe loaded nanoparticles to monitor the real-time tumor-targeting ability of the DDS. The distribution of DDS at the tumor tissue was reflected by monitoring the NIR fluorescence within the tumor site of HepG2 tumor-bearing nude mice for up to 6 h (Figure 5A). The *in vivo* images at the tumor site after intravenous injection of DiR-loaded nanoparticles at different time points were recorded. It was clear that SLN/DTX/TDD and LDL/SLN/DTX/TDD showed significant differences in targeting efficacy after *in vivo* administration as the fluorescence intensity of tumor site in LDL/SLN/DTX/TDD-treated mice was stronger than that of SLN/DTX/TDD at the same period.

The difference in tumor targetability was also verified by using *ex vivo* imaging of the tumor tissues and main organs of the sacrificed mice (Figure 5B). In detail, the fluorescence intensity of the tumor tissues in the LDL/SLN/DTX/TDD-treated mice was 5.21-fold higher than the SLN/DTX/TDD-treated mice. Besides, it was interesting to find that the fluorescence intensity of the livers showed an opposite result as in the SLN/DTX/TDD-treated mice it was 3.32-fold higher than the LDL/SLN/DTX/TDD-treated mice. These results indicated that LDL/SLN/DTX/TDD had stronger tumor-targeting ability than SLN/DTX/TDD as the former showed preferable accumulation at the tumor tissue. This was beneficial for LDL/SLN/DTX/TDD as a safe and effective DDS to deliver their load to the targeted cells and achieve better anti-cancer effect.

### *In vivo* anti-cancer efficacy

**Figure 6.**
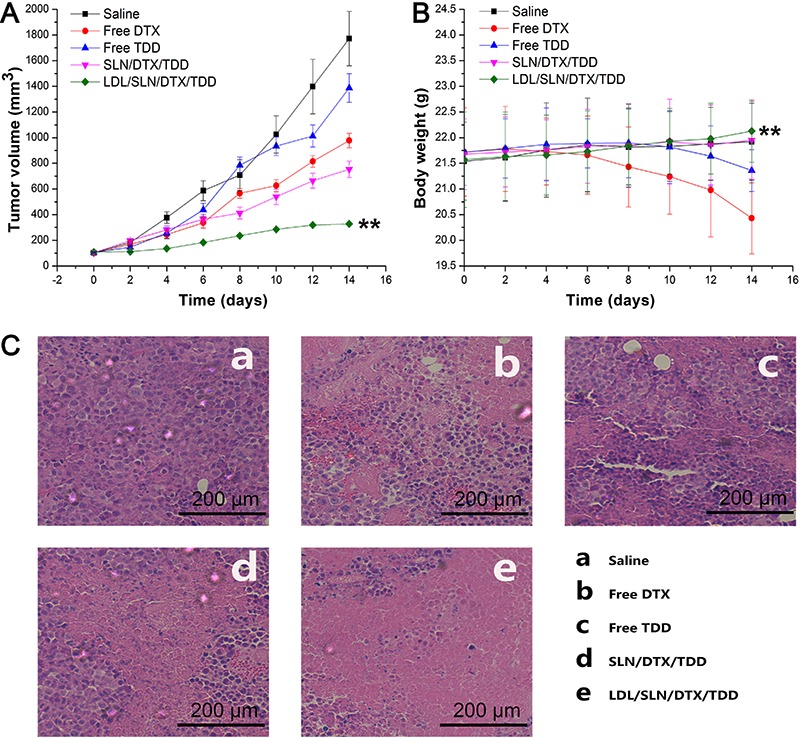
Tumor volume (*A*) and body weight (*B*) of the different groups. *C*, HE staining of tumor tissue of HepG2 tumor-bearing BALB/c nude mice after intravenous injection of different formulations. The measurement of tumor volumes and the injection of formulations were repeated every 2 days for 2 weeks. Dose: 10 mg/kg DTX and/or 25 mg/kg TDD per mouse. LDL: low density lipoprotein; SLN: silica nanoparticles; DTX: docetaxel; TDD: thalidomide. Data are reported as means±SD (n*=5*3). **P<0.01 *vs* saline (panel *A*); **P<0.01 *vs* Free DTX (panel *B*) (two-tailed Student's *t-*test).

With the aim to seek the *in vivo* antitumor potential of LDL/SLN/DTX/TDD in HepG2 xenografted nude mice, LDL/SLN/DTX/TDD as well as SLN/DTX/TDD and free drugs (DTX and TDD) were assessed concerning their ability to suppress tumor growth and influence body weight variation with saline as a blank control. As shown in Figure 6A, it was interesting to note that the anti-cancer effects of LDL/SLN/DTX/TDD and SLN/DTX/TDD were significantly better than free drugs at the same drug concentrations. The opposite results to the cytotoxicity assay indicated that DDS improved the anti-cancer efficacy of free drugs. Furthermore, although all the formulations suppressed tumor growth to some extent, the anti-cancer efficacy of LDL/SLN/DTX/TDD appeared to be most potent compared with others since animals treated with LDL/SLN/DTX/TDD showed the smallest tumor volumes of 328 ± 43 mm^3^. The observations suggested the enhanced tumor-homing property of LDL/SLN/DTX/TDD due to the modification of LDL.

Moreover, the time- and formulation-dependent variation in body weight of subjected mice was recorded. Free drugs, especially DTX, demonstrated to have strong system toxicity, which would result in body weight loss as shown in Figure 6B. The body weight of mice treated with free DTX steadily decreased since day 4, while the TDD-treated mice began to lose weight at day 10, indicating that the health condition of mice was compromised either due to tumor burden, side effects of free drugs or their combination. However, no noticeable loss in body weight was observed in LDL/SLN/DTX/TDD-treated group, suggesting that the DDS could not only increase the anti-cancer efficacy of free drugs, but also reduce its risks.

The tumor tissues from mice treated with three different formulations were subjected to HE staining and representative images are displayed in Figure 6C. It was observed that cells in the negative control group (saline) showed typical pathological characteristics of tumor, such as large and irregularly shaped nuclei closely packed with one another. Other drug formulations showed characteristics of cancer cell remission, with tumor coagulative necrosis, intercellular blank and nuclei fragmentation being observed. Compared with the other groups, LDL/SLN/DTX/TDD group showed a massive cancer cell remission with the most promising antitumor ability, consistent with the above-mentioned results.

In summary, a tumor-targeting DDS composed of LDL and SLNs loaded with DTX and TDD was developed (LDL/SLN/DTX/TDD) to take advantage of the tumor-targeting ability of LDL and drug-loading property of SLNs for potential chemotherapy of liver cancer. Our experimental results indicated that nano-sized LDL/SLN/DTX/TDD with decent drug loading could preserve the encapsulated drugs under physiological conditions while releasing drugs faster under acidic neoplastic conditions. On the other hand, LDL/SLN/DTX/TDD can increase the uptake ratio of drugs into HepG2 liver cancer cells compared with unmodified SLN/DTX/TDD, possibly via the LDLR-mediated endocytosis. More importantly, LDL/SLN/DTX/TDD exhibited stronger anti-cancer activity *in vivo* via the synergetic effect of DTX and TDD with minimized toxic side effects and preferable tumor-suppression potential on tumor-bearing nude mice model.
